# Interspecific grafting between *Gossypium hirsutum*, *G. barbadense* and *G. herbaceum* lines

**DOI:** 10.1038/s41598-020-75679-1

**Published:** 2020-10-29

**Authors:** Mehmet Karaca, Ayse Gul Ince, Umesh K. Reddy

**Affiliations:** 1 Department of Field Crops, Faculty of Agriculture, Akdeniz University, 07059 Antalya, Turkey; 2 Vocational School of Technical Sciences, Akdeniz University, 07059 Antalya, Turkey; 3 Department of Biology, West Virginia State University, Institute, WV 25112 USA

**Keywords:** Biological techniques, Plant sciences

## Abstract

Seedling grafting could provide additional crop improvement strategies for cotton. However, there existed limited studies on interspecific grafting and approaches. Four different grafting approaches were developed and compared between lines representing three of the four cultivated cotton species *G*. *hirsutum*, *G. barbadense* and *G. herbaceum*. Grafting approaches of this study focused on the cotyledon node and cotyledon leaves retained on scions, rootstocks, without cotyledon node and cotyledon leaves on scions and rootstocks or halved cotyledon node and single cotyledon leaf on scions and rootstocks. Evaluations of the grafting approaches were made by comparing survival and growth rate during the second and fifth weeks after transplantation, respectively. The formation of any lateral shoots at the grafted sites were studied in two of four grafting approaches in the first and the second year during flowering stage. DNA alterations due to grafting were investigated using microsatellite markers. There were no statistically significant differences between grafts and their control in survival rate and locus specific DNA alteration. Growth rate and lateral shoot formation, on the other hand, were different among grafting types and grafts. We concluded that grafting without cotyledon node and cotyledon leaves on rootstocks, and with cotyledon node but without cotyledon leaves on scions were easy to perform and suitable for interspecific cotton grafting. Results suggested that grafting seedlings and allowing time to heal graft wounds prior to spring transplanting or double cropping is suitable for wheat–cotton intercropping to prevent late or early chilling damage associated with seed sowing or conventional transplanting of susceptible seedlings. Furthermore, the rapid and consistent wound healing in seedling grafts along with lateral shoot formation occurring in two of four grafting approaches make them a suitable approach to investigate possible genetic and epigenetic movement between scions and rootstocks, especially across species.

## Introduction

The genus *Gossypium* L. contains more than 50 species, four of which are cultivated in the world. These four cotton species include *Gossypium arboreum* L., also known as “Tree Cotton”, native to the Indian subcontinent, *G. barbadense* L., also known as “American Pima”, “Egyptian Cotton” or “Sea Island Cotton”, native to tropical South America and the Nile region, *G. herbaceum* L., known as “Levant Cotton”, native to southern Africa and the Arabian Peninsula, and finally *G. hirsutum* L., known as “Upland Cotton”, native to Central America. Among the cotton species, *G. hirsutum* is the most widely cultivated species worldwide^1,2^. Annual fiber production and plantation area of cotton vary from year to year but the most recent data showed that fiber production was about 26 million tons and planted area was about 35 million hectares^3,4^. Among the cotton-producing countries, India, the People’s Republic of China, the United States of America (USA), Pakistan, Uzbekistan and Brazil are the leading countries. Turkey and Greece are the main cotton producers in Europe but Spain and Bulgaria produce some amount of cotton^3^.

Interspecific crossing for crop improvement experiments within and between major species, including cotton produced limited success^2,5^. In traditional agriculture of cotton, grafting, transplantation and pruning practices were not widely used^4^. However, these practices have been increasingly applied in some areas of China and with the need for more rotational cropping and transplanting of seedlings, it seems that there will be a wider application of these practices in cotton agriculture^4,6^. Grafting is a vegetative propagation event that occurs spontaneously in nature or artificially assisted by humans^7^. In vegetative grafting experiments, the shoot part of a plant (known as scion) is attached onto a root part of another plant (known as rootstock). Majority of grafting experiments are undertaken to improve biomass accumulation, fruit quality, and provide resistance to biotic stresses such as soil-borne pests and diseases, to increase tolerance to acidity, water deficit, salinity and other abiotic environmental stresses^7–10^. Although there exist various hypotheses on the molecular mechanism underpinning grafting responses, we still know little of how grafting with rootstocks confers differences to the vigor of the scion or vice versa.

Previous studies have shown that success of grafted plants (compatibility) was dependent on the type of environment (and ambient factors), species, genotype, developmental stage and growth processes^10^. In cotton, grafting experiments have been conducted for different purposes including transfer of root-knot nematode resistance^11^, recovery of plants from in vitro culture^12^, transgenic recovery^13^, transfer of cytokinins and abscisic acid hormones^14^, transfer of *Verticillium dahlia* resistance^15^, identification of the role of shoot on premature leaf senescence induced by potassium nutrition^16^, increased cryotolerance^17^, and resistance to leaf curl disease^18^. In a study of grafting experiments, Zhoa et al.^19^ concluded that the major source of gossypol was root system in glanded and glandless cotton. However, our unpublished data does not confirm this finding instead; we found that gossypol is also synthesized from other tissues in cotton.

In plants, several grafting techniques such as side, bark, saddle, bridge, inarch, splice and mentor grafting methods are being used. However, most of these techniques are not suitable for cotton. Grafting methods of modified cleft and wedge seem suitable and have been used in several grafting methods including micro-grafting and seedling-grafting^12,16,20,21^. Micro-grafting of cotton uses shoot apex (micro-shoot) from a donor plant and joined onto a young decapitated plant grown in greenhouse/growth chamber under aseptic growth conditions^20^. In a modified cleft-graft method described in Luo and Gould^12^, shoot immediately above the cotyledon node is removed while keeping cotyledon leaves. The node-hypocotyl axis is split vertically to a depth of 2–4 cm. Shoot tip scion taken from culture is cut to form a deep ‘V’. The scion and rootstock are joined vertically and secured. In vitro and ex vitro micro-grafting of cotton have been developed for micro-shoots of *G. hirsutum*. However, ex vitro micro-grafting had very limited survival rate (30%) and in vitro grafting required longer time for obtaining mature plants and had 70% survival rate. Furthermore, the requirement of tissue culture techniques and etiolation make this method time-consuming and a more expensive approach^20^. Jin et al.^13^ reported an in vitro grafting procedure that was principally very similar to micro-grafting reported in Banerjee et al.^20^. In another study, Li et al.^16^ developed three approaches, one of which was called standard grafting involving one scion and one rootstock, the second type was called “Y” grafting with two scions grafted onto one rootstock and the third type was called inverted Y grafting with one scion grafted onto two rootstocks. Regardless of the scion and rootstock numbers, in all three grafting approaches, scions and rootstocks were joined at the cotyledon node by the wedge-grafting technique.

This study was undertaken to develop and compare four modified cleft-wedge-grafting approaches between three cotton lines [Texas Marker-1 (TM-1), Pima 3–79 and Maydos Yerlisi (MY)] representing three *Gossypium* species (*G. hirsutum*, *G. barbadense* and *G. herbaceum*, respectively) cultivated worldwide to determine an efficient grafting method that could be used in cotton. Efficient grafting means proportional growth of rootstock and scion, no retarded growth in comparison to control (not grafted) seedlings, no lateral shoot formation from the graft junction of rootstocks and without DNA alteration in the grafted plants using microsatellites, also known as simple sequence repeats (SSRs).

## Results

We noted that when suitable humidity (about 80%) and temperature (28 °C) provided to scions attached on rootstocks in growth chamber or tunnel, scions could survive more than 1 week without any dying symptoms even incompatible intergeneric grafting as we noted in grafts between cotton and sunflower. Therefore, survival rate of grafting was determined at the fourth week of grafting experiments (two weeks after transplanting). Expanding the true leaves on scions and emergence of new leaves from the terminal buds of scions were considered as success of grafting. Growth rate was another parameter we used to assess the differences between the grafting approaches. The number of leaves on the grafts along with the control seedlings were counted five weeks after transplantation (seventh weeks after grafting). Healthy and actively growing grafts produced more leaves indicating higher growth rate. Controls showed higher growth rate than all grafts probably due to wounding effects on the grafts. Third evaluation parameter was lateral shoot formation on graft junction of rootstock. Although this parameter is not directly related with the graft compatibility, it is very important for homogeneity of graft products, for instance, fiber technical quality characteristics^2^. Most studies utilize rootstock diameter, scion diameter, scion length, rootstock length, number of leaves in tree grafting experiments^22–24^. We noted that two parameters, survival and growth rate, were suitable for identification of grafting success in cotton.

In interspecific grafting, the second most important aspect after the humidity and temperature was the physiological stage of scions and rootstocks. Our initial studies showed that survival rate of grafting approaches I, II, and III were low when the number of leaves of seedlings were fewer than two (at very young stage) or more than eight leaves (older stage). On the other hand, grafting approach IV produced successful grafting even when seedlings used were with leaves in emerging stage. Grafting approaches, I, II and III could be successfully completed within one month from sowing to field transplantation while type IV required one week less time duration. Our initial studies also revealed that date between sowing to transplantation could be extended up to 2 months allowing the seedlings grow further in a greenhouse or a growth tunnel before field transplantation. This would allow us to sow cottonseeds two months before winter-wheat harvest. In another words, this means that planting time of cotton could be expanded two months earlier in winter-wheat-cotton intercropping production system if grafting followed by transplanting is used^6,25,26^.

In the present study, plant survival after transplantation was checked for two years in a greenhouse, none of the grafted plants showed incompatibility indicating that all the three cotton species were highly graft compatible. It is known that grafts within the same genus were always compatible when similar size rootstocks and scions were used. However, when rootstock and scion belong to different family the grafting was not successful. In the present study, none of the four grafting approaches was successful between *G. hirsutum* and *Helianthus annuus*, as previously reported^19^. Previous studies revealed that intra familial grafts are rarely compatible, and inter familial grafts are essentially always incompatible^27^. It is known that taxonomic relationship is a general prerequisite for successful grafting rate and survival rate of the grafted plants^7^.

### Grafting type I

**Figure 1:**
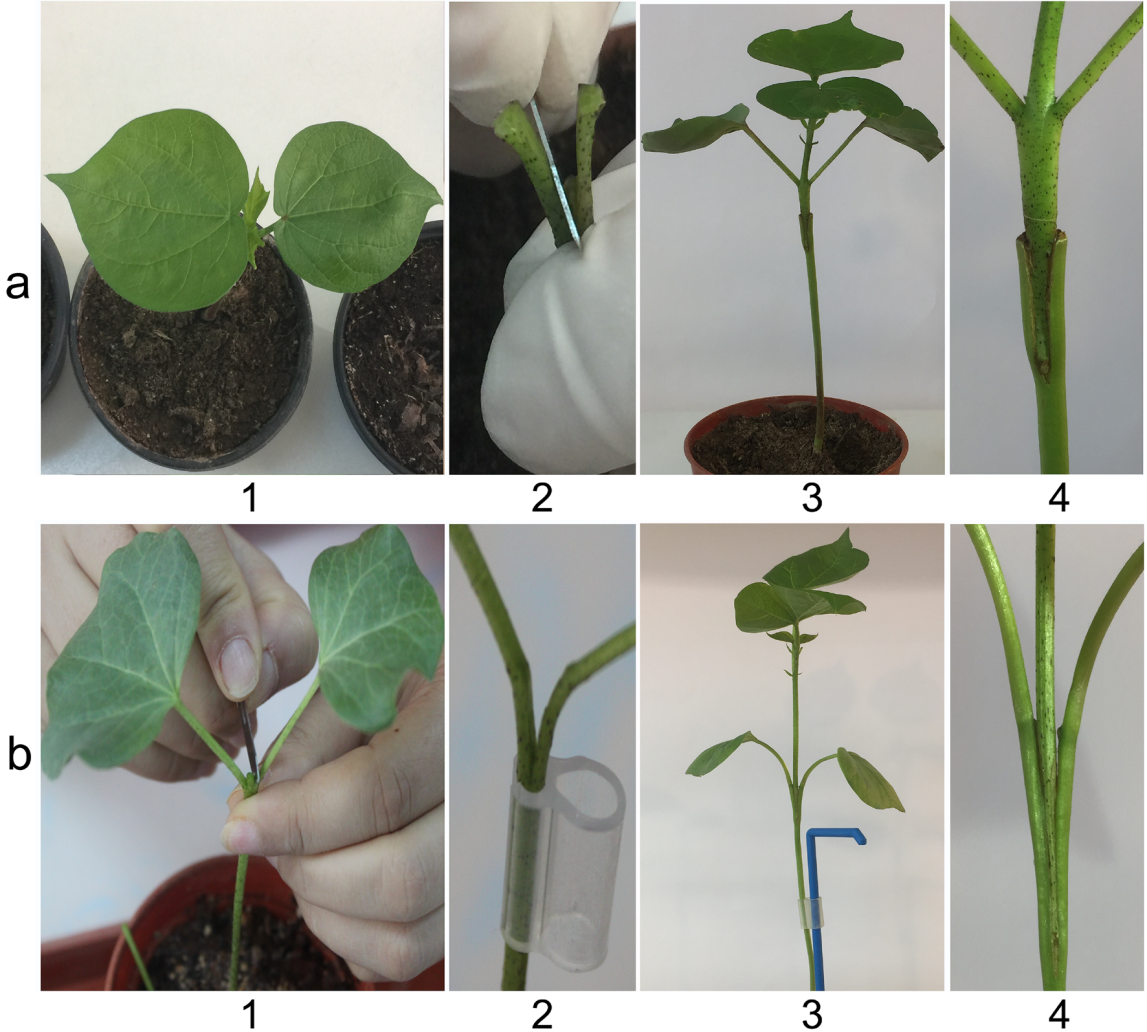
Representation of various stages of grafting type I and II and seedlings of scions and rootstocks. (**a**) Type I, a1: a rootstock seedling; a2: cutting vertically downward to a depth of 2–3 cm to have a deep ‘V’ shape; a3: a graft after 15 days of grafting and a4: scion and rootstock joint junction of a graft after 15 days of grafting and; (**b**) Type II, b1: cutting vertically at the cotyledon node between the cotyledon leaves; b2: prepared rootstock; b3: a graft after 15 days and b4: scion and rootstock joint junction of a graft.

This type of grafting consisted of rootstocks that had no cotyledon node and cotyledon leaves while scions contained cotyledon node and cotyledon leaves. In this type, as shown in Fig. 1a, scions with cotyledon node and cotyledon leaves were inserted onto rootstocks in a fashion that deep V cut ends firmly joined. Sowing to transplantation took about one month in this type of grafting. Survival rate (Table 1), growth rate (Table 2), lateral shoot formation (Table 3) and locus specific DNA alteration were used to compare this approach with the other three grafting approaches developed in this study. Survival rate was not statistically significant at the α = 0.05 level among the nine different interspecific grafting. In addition, there were no interactions between grafting approaches and grafts (Table 1).

**Table 1:** ANOVA and Tukey–Kramer means separation of seedling survival rate according to grafting type and species of *Gossypium*, with rootstock–scion combinations presented in this exact order.

Analysis of variance for survival rate					
Source	DF	Sum of squares	Mean square	F ratio	Prob > F
Model	47	6.22	0.13	0.68	0.9273
Error	96	18.67	0.19		
C. Total	143	24.89			

Source	DF	Sum of squares	F ratio	Prob > F	
Graft	11	2.72	1.27	0.2519	
Grafting	3	0.17	0.28	0.8356	
Graft*Grafting	33	3.33	0.52	0.9826	

Graft		Tukey–Kramer HSD groupings*		Mean (seedling no)	
Pima 3–79 (Control)		A		4	
TM-1 (control)		A		4	
MY (control)		A		4	
[TM-1—Pima 3–79]		A		3.83	
[MY—MY]		A		3.75	
[TM-1—TM-1]		A		3.75	
[MY—Pima 3–79]		A		3.67	
[MY—TM-1]		A		3.67	
[Pima 3–79—MY]		A		3.67	
[Pima 3–79—Pima 3–79]		A		3.67	
[Pima 3–79—TM-1]		A		3.67	
[TM-1—MY]		A		3.67	

Grafting		Tukey–Kramer HSD groupings*		Mean (seedling no)	
Type III		A		3.83	
Type II		A		3.78	
Type I		A		3.75	
Type IV		A		3.75	

*Levels not connected by the same letter are significantly different (α = 0.05).

**Table 2:** ANOVA and Tukey–Kramer means separation of seedling growth rate according to grafting type and species of *Gossypium*, with rootstock–scion combinations presented in this exact order.

Analysis of variance for growth rate						
Source	DF	Sum of squares	Mean square	F ratio	Prob > F	
Model	47	79.56	1.69	3.69	< 0.0001*	
Error	96	44.00	0.46			
C. Total	143	123.56				

Source	DF	Sum of squares	F ratio	Prob > F		
Graft	11	67.72	13.43	< 0.0001*		
Grafting	3	4.17	3.03	0.0331*		
Graft*Grafting	33	7.67	0.50	0.9856		

Graft	Tukey–Kramer HSD groupings*					Mean (leaf no)
Pima 3–79 (Control)	A					5.5
TM-1 (Control)	A					5.5
MY (Control)	A	B				5.25
[Pima 3–79—Pima 3–79]	A	B				5
[TM-1—TM-1]	A	B	C			4.83
[TM-1—Pima 3–79]	A	B	C			4.83
[MY—Pima 3–79]		B	C	D		4.5
[Pima 3–79—TM-1]		B	C	D		4.41
[MY—TM-1]			C	D	E	3.91
[MY—MY]				D	E	3.75
[Pima 3–79—MY]				D	E	3.75
[TM-1—MY]					E	3.41

Grafting	Tukey–Kramer HSD groupings*					Mean (leaf no)
Type I	A					4.8
Type IV	A	B				4.6
Type II	A	B				4.5
Type III		B				4.3

*Levels not connected by the same letter are significantly different (α = 0.05).

**Table 3:** ANOVA and Tukey–Kramer means separation of lateral shoot formation according to grafting type and species of *Gossypium*, with rootstock–scion combinations presented in this exact order.

Analysis of variance for lateral shoot formation					
Source	DF	Sum of squares	Mean square	F ratio	Prob > F
Model	47	19.31	0.41	4.55	< 0.0001*
Error	96	8.67	0.09		
C. Total	143	27.98			

Source	DF	Sum of squares	F ratio	Prob > F	
Graft	11	3.97	4.00	< 0.0001*	
Grafting	3	10.91	40.31	< 0.0001*	
Graft*Grafting	33	4.42	1.48	0.0719	

Graft	Tukey–Kramer HSD groupings*				Mean (lateral shoot no)
[MY—Pima 3–79]	A				0.42
[Pima 3–79—MY]	A				0.42
[Pima 3–79—TM-1]	A				0.42
[TM-1—Pima 3–79]	A				0.42
[MY—MY]	A				0.42
[Pima 3–79—Pima 3–79]	A	B			0.33
[MY—TM-1]	A	B			0.25
[TM-1—MY]	A	B			0.25
[TM-1—TM-1]	A	B			0.25
MY (Control)		B			0
Pima 3–79 (Control)		B			0
TM-1 (Control)		B			0

Grafting	Tukey–Kramer HSD groupings*				Mean (lateral shoot no)
Type IV	A				0.64
Type II		B			0.42
Type I			C		0
Type III			C		0

*Levels not connected by the same letter are significantly different (α = 0.05).

There was statistically significant difference at the α = 0.05 level between growth rate of type I grafting and type III grafting but it was not statistically significant when comparing this type with type II and IV. However, growth rate differences were observed between controls (Pima 3–79, MY and TM-1) and their grafts (Table 2). In addition, there were no interactions between grafting approaches and grafts (Table 1). Number of leaves were less on seedling grafts due to graft wounding effect and vascular reformation, retarding the growth^28^, however, we noted that growth rate retardation was not detectable later around the flowering stage.

The main disadvantage of this grafting type was encountered when tetraploid species were used as scions onto rootstocks of diploid species. In these grafts, a number of scions lost their cotyledon leaves probably due to the insufficient water and nutrient transfer from rootstock^29^. However, the loss of cotyledon leaves after grafting did not severely affect the survival rate but there was some level of negative effect on growth rate probably due to wounding^28^. During the two-year observations, we did not note the presence of lateral shoot formation in grafts of this grafting approach (Table 3).

In the present study, SSR marker analysis indicated that there was no locus-specific DNA alteration in the seeds of grafts due to the grafting. It is accepted that grafting does not alter the genetic content of the graft partners and their offspring. However, there are considerable amount of studies reported the DNA alteration due to grafting^9,30–34^. Previous studies also showed that genetic material could be transported via plasmodesmata between stock and scion cells in interspecific graft junction zones between different *Nicotiana* species^35^. In the present study, our experiments used just 10 loci and seeds only, therefore; more extensive and detailed research is needed to fully determine if any of our grafts experienced graft induced genetic alteration or exchange.

### Grafting type II

Scions of this type grafting were inserted at the cotyledon node of rootstocks (Fig. 1b). Compared to type I, inserting and wrapping the graft joint was a little bit difficult due to further attention required not to damage cotyledon node and cotyledon leaves. The use of cotyledon node at the cut site of grafting allowed us use of relatively thicker diameter of the shoots present at the node. Although sowing at different date could help us to obtain similar shoot diameter among seedlings of different species, this type of grafting was especially advantageous in grafting of *G. herbaceum* (rootstocks)—*G. barbadense* and *G. hirsutum* (scions) where there exist greater shoot diameter differences between graft partners. Stem diameter differences among the three cotton species were the most affected from temperature and light quality.

Survival rate was not statistically significant between type II grafting and other three grafting approaches (Table 1). However, there were significant growth rate differences between grafts and their controls (Table 2). Analysis revealed that there was statistically significant difference at the α = 0.05 level between lateral Type II grafting produced lateral shoots (Fig. 3) which was disadvantageous because the presence of lateral bud produced shoots should not be allowed because they reduced the purity of cotton fiber technical characteristics. Observations revealed that induction of lateral shoot formation often occurred on the rootstocks; *G. hirsutum*, followed by *G. barbadense* and less frequent in *G. herbaceum*. Results of the present study revealed that cutting at the cotyledon node or damaging the cotyledon leaves of rootstocks induced the formation of lateral shoots probably removing the dormancy of two buds at the cotyledon nodes due to the hormonal regulations^28^. We also noted that there was no locus specific DNA alteration between type II grafting and control seedlings.

### Grafting type III

**Figure 2:**
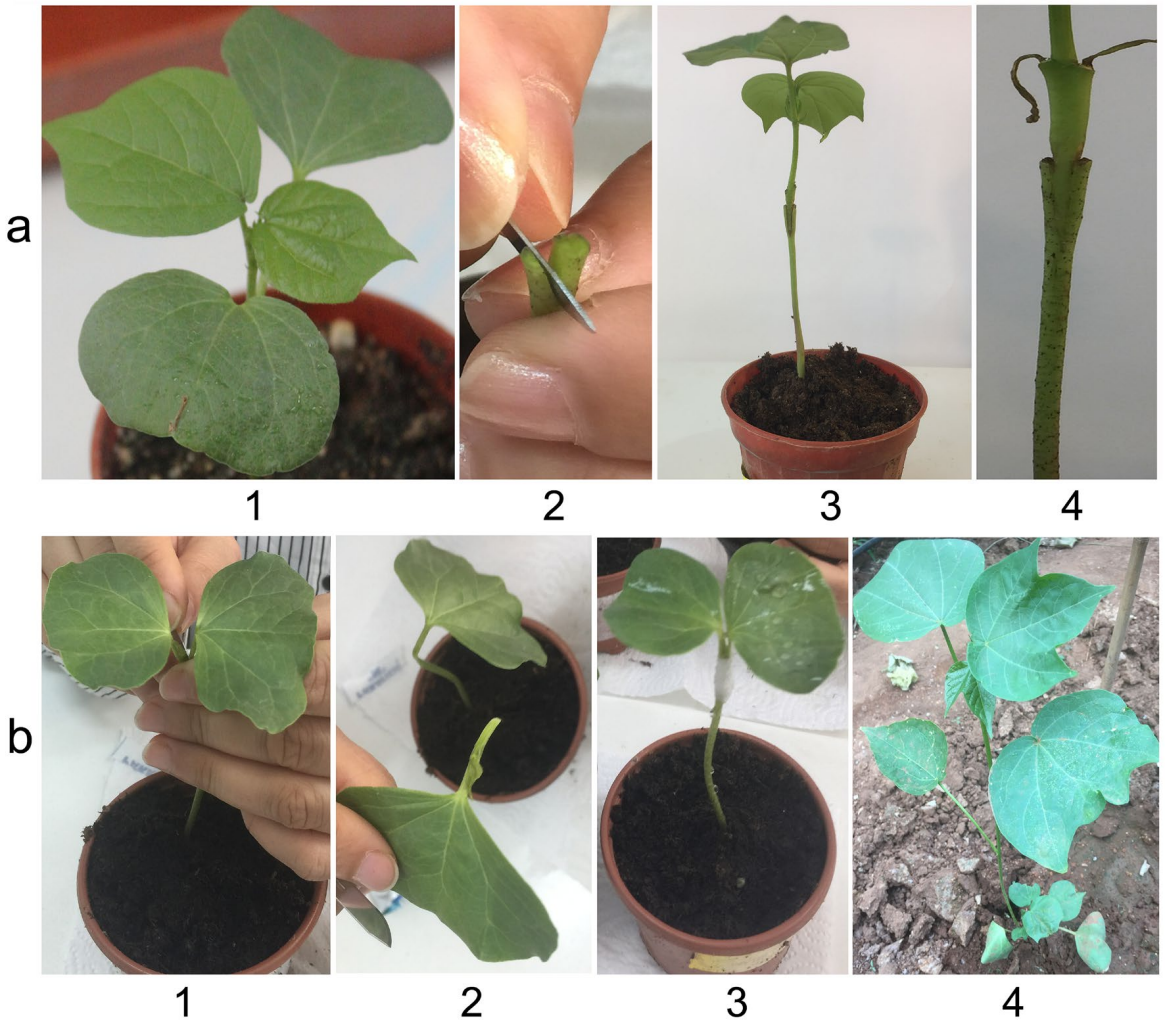
Representation of various stages of grafting type III, IV, and seedlings of scions and rootstocks. (**a**) Type III, a1: a scion seedling; a2: ‘V’ shape cutting; a3: a graft after 15 days of grafting and a4: scion and rootstock joint junction of a graft; (**b**) Type IV, b1: cross cutting at cotyledon node; b2: removing the half cotyledon node and single cotyledon leaf; b3: a graft just made, b4: a graft with a developing lateral shoot after 2 weeks of transplantation.

In this type grafting, cotyledon node and cotyledon leaves on rootstocks were removed by cutting with a razor blade. However, scions contained cotyledon node but not cotyledon leaves (Fig. 2a). We noted that finding suitable seedlings that were used as scions with similar diameter above the cotyledon node and shoots of rootstocks for this approach was difficult in cotton seedlings, probably due to genomic and physiological differences among lines of *G. hirsutum*, *G*. *barbadense* and *G*. *herbaceum*. Cotton seedlings usually show characteristically wider stem diameter around the cotyledon nodes. Therefore, keeping 2–3 cm of shoots above the cotyledon nodes on the scions was very suitable for interspecific grafting between tetraploid (as rootstocks) and diploid species (as scions).

There were no statistically significant differences between survival rate (Table 1) of this grafting type and other three grafting approaches. However, growth rate of type III grafting approach was statistically different from type I grafting (Table 2) indicating that presence of cotyledon leaves on scion was beneficial for better growth. Statistically significant differences among grafts and their controls were found in this approach (Table 2) but there were no statistically significant interactions between grafts and grafting types. There were no significant differences on the lateral shoot formation between type III and type I grafting but significant differences existed between type III and type IV, between type III and type II (Table 3). We noted that there were no locus specific DNA alterations between grafts obtained using this approach and their controls.

### Grafting type IV

In the type IV grafting approach, both scions and rootstocks contributed a half cotyledon node attached with one cotyledon leaf. In another words, a cotyledon node and its leaf were removed from both the scion and rootstock prior to grafting (Fig. 2b) and were joined. There were no differences between survival rates of this type grafting in comparison to other three approaches (Table 1). In addition, growth rate was not statistically different between type IV and other grafting approaches (Table 2). On the other hand, lateral shoot formation significantly differed between type IV and type II, between type IV and type I, and between type IV and type III (Table 3) grafting approaches. We did not note any locus specific DNA alterations in this grafting approach.

This type grafting was difficult to make because it needed seedlings that were used as scions and rootstocks with very similar developmental stage and shoot diameter. Furthermore, because both lateral buds from scions and rootstocks at the cotyledon node induced by cuttings, the lateral shoot formation of this grafting approach was much higher among grafting approaches used in this study. However, the use of this grafting approach reduced the required time between sowing and grafting, and between grafting and transplantation to field. Greenhouse-grown seedlings with two true developing leaves were used as rootstocks and scions in grafting type I, II and III while seedlings with emerging the first leaf were utilized in grafting type IV.

**Figure 3:**
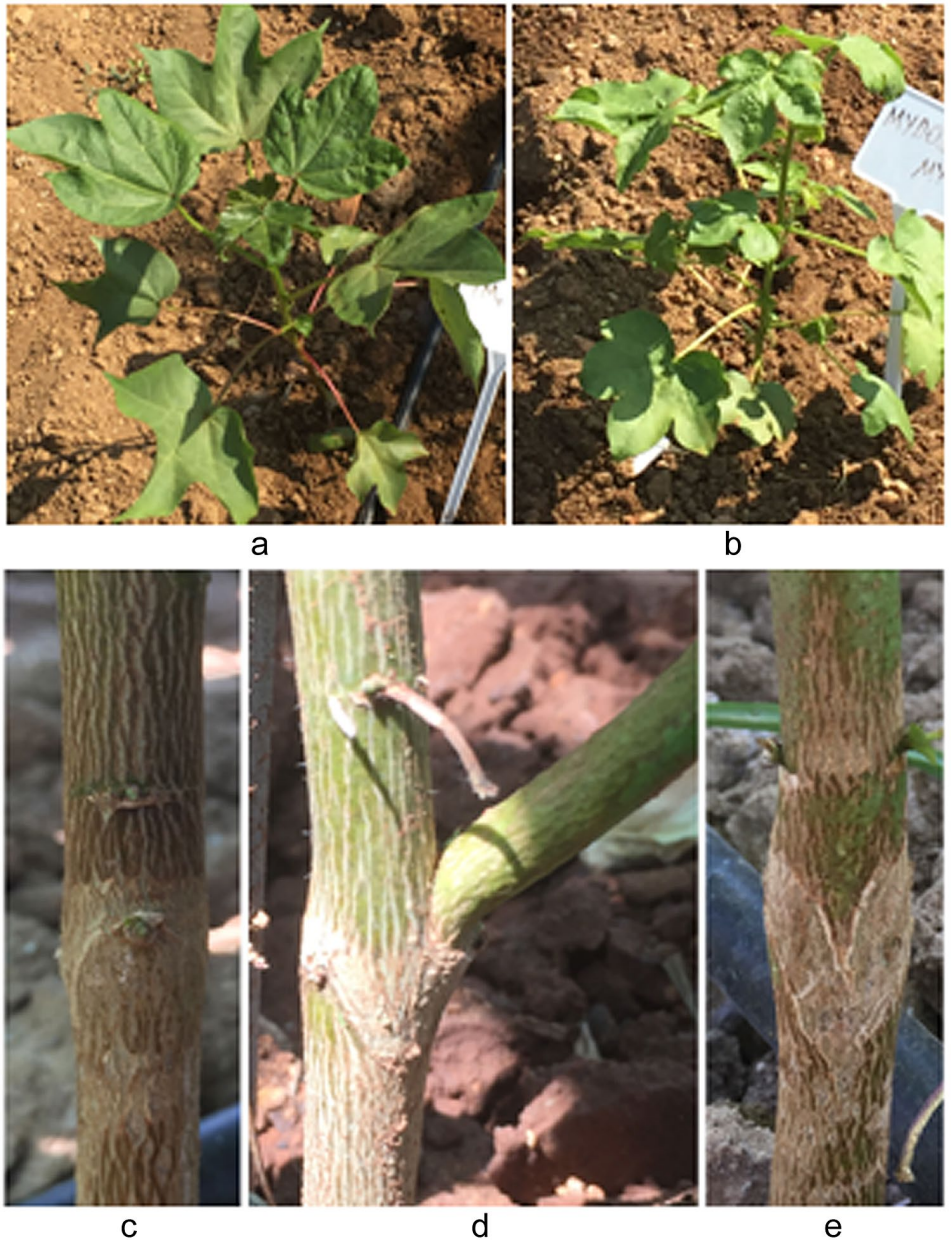
Representation of various development stages and graft junctions of mature grafts. (**a**) Filed growing grafts: [TM-1—Pima 3–79], (**b**) [MY—MY], (**c**) [TM-1—Pima 3–79], (**d**) lateral shoot formation on TM-1 rootstock of graft [Pima 3–79—TM-1] and (**e**) [Pima 3–79—Pima 3–79].

In all the grafting approaches used in the present study, grafts were under grafting shock due to wounding effects, which took 4–7 days for cell divisions at the graft junction sites. After transplantation to a greenhouse or a field, all seedling grafts were scanned to check proper formation of graft joints and the presence of lateral shoots. At the flowering stage, graft unions were investigated to confirm the long-term graft compatibility. As shown in Fig. 3, scion and rootstock of grafts joined perfectly in both greenhouse grown and field grown plants indicating the compatibility. Although the time requirement of wound healing differed among the cotton lines representing three of the four cultivated cotton species, we did not observe any grafting incompatibility among all the combination of intraspecific and interspecific grafting studies.

Among all grafting approaches used in the present study, we did not detect any apparent dramatic phenotypic changes such as dwarfing and differences of scion-rootstock stem diameter growth or irregularity, which could indicate incompatibility between scions and rootstocks. In some crops for instance in apple, nutrient and water movement, hormone concentrations and anatomy of graft union, some biochemical changes were altered^22–24,36–41^. Grafting induced phenotypic changes have been reported in a study of Li et al.^10^, in which two chimeras of tuber mustard and red cabbage were used to study the mechanisms and the inheritance of the variation induced by grafting. Their results revealed that grafting caused changes in leaf shape and the pattern of shoot apical meristem termination. However, in our studies we did not detect any phenotypic variation due to grafting effects.

## Discussion

We noted that ambient humidity and temperature after grafting experiment were very two important factors for successful interspecific grafting studies. Selection of healthy and physiologically similar seedlings especially similar diameters for grafting experiments was also important aspects in interspecific cotton grafting. Grafting approaches used in the present study did not severely affect survival rate and caused DNA alteration, but much differed in growth rate and lateral bud formation. Higher survival rate of seedling among all four grafting approaches was probably due to the fact that seedlings have lower transpiration rates and probably heal better with grafting than older tissues with rapid transpiration rates. The ability to recombine high quality and yield in cotton with critical resistances to biotic and abiotic stresses in more exotic germplasm is very difficult. Therefore, seedling grafting with higher survival rate could be an instant way to combine superior rootstocks with scions of elite cotton cultivars. Perhaps transgenic rootstocks will be easier to use with little to no danger of genetic spread through seed^13^. If the rootstock has a marker trait like red stems or leaves then it could be used as a genetic marker to remove any incidence of lateral shoot formation. However, despite its long history and practical utilities of plant vegetative grafting, the underlying mechanisms for graft-induced phenotypic and physiological changes remains to be fully understood. It is generally accepted that metabolic substances including those that may produce large biological effects, such as hormones, proteins and signal molecules, could be transferred from one grafting partner to the other^18,28,38,42^ causing changes in graft partners. Also healing response is known to involve in salicylate^36,37^, jasmonate^38^, and other plant hormone signals^39,40^. The plant genotype and whether the tissues have cotyledon node and leaves may affect the synthesis of key compounds involved in successful graft healing and transmission. Grafting approaches developed in this study could be used to enhance our knowledge on grafting phenomenon.

Statistically significant differences between growth rates of grafted seedlings and their controls may be a result of some grafts (type I versus type III) retaining the presence of cotyledon node and cotyledon leaves on scions which enhanced the initial accumulation of photosynthetic products. The cotyledon node and leaves have additional significance if left on the rootstock (type II and IV) because of lateral shoot formation from wounding due to cutting the graft zone. Logically we expect that both tissues of the graft may synthesize the optimal compounds for rapid graft healing and transmission with keeping one of its own cotyledon node and leaf.

Results clearly showed that differences in growth rate due to the wound healing and vascular regeneration within the graft union zone were not affected with the grafting approaches; therefore, we speculated that retardation on the growth rate was probably due to hormonal signals such as auxin and cytokinin^7,8,28,40^. The effects of wounding on growth rate were clearly observed on the growth rates between grafts and their controls. When seedlings reached to flowering stage the differences between the grafted plants and their control were not visible, however, the onset of flowering was different depending on the species and intraspecific and interspecific grafting. We are carrying further experiments on graft-induced alteration on fiber technical properties of cotton.

We noted that not all grafts produced lateral shoots probably due to the damage to very delicate axillary buds occurred during the preparation of rootstocks or different level of hormones in different species or physiological states of the tissues, presented in the rootstocks, which can either suppress or initiate axillary bud growth. Adverse effect of wounding and callus formation is relatively less with seedling grafting than with older and larger tissues. In addition, the balance between size and age between scion and rootstock is better with seedling grafting. Furthermore, grafting with seedlings seems a better way to start with virus free plants as older plants can get and transmit viruses from being exposed to insects. This can be critical for some cotton production areas.

Grafting approaches reported here could be used in cotton improvement studies. For instance, interspecific grafting experiments would allow the increase in some physiologic, agronomic, genetic and epigenetic traits^34,42^. Grafting experiments have been already used in cotton for different purposes. Some of these experiments resulted in alteration of traits that have biological and economical values. For instance, Li et al.^16^ reported change in the boll weight (g) at different lateral fruit positions of sympodial branches among scions/rootstocks. Dong et al.^14^ found that the graft of early senescence scions onto late senescence rootstocks alleviated leaf senescence, whereas that of late senescence scions onto early senescence rootstocks enhanced leaf senescence. Thus, they inferred that leaf senescence was considerably affected by the root genotype. Some other grafting effects in cotton have been previously reported including the gain of increased cryotolerance and the overwintering survivorship ^17^, resistance to leaf curl disease ^18^, *Verticillium dahlia* resistance^15^, yield and fiber quality. A study by Hao et al.^34^ provided evidence of horizontal gene transfer events via graft transmission in cotton. The consistent and high quality grafts produced in our study could be a suitable model to further study chemical and genetic changes and transmission between scion and rootstock, especially when using diverse species of cotton.

In the present study, we noted that growth rate not directly related to survival rate but it could be important for the elapsed time between seed sowing to transplantation of grafted plants. Increased growth rate related with earliness, which is important for double cropping within a year. Cotton growing in most of the countries as in Turkey is dependent upon limited irrigation especially during dry seasons and low temperature during the early spring and low temperature along with precipitation in late fall. Drought or cold stress in spring often causes delay in cotton-planting date for the single crop production system that involves planting in mid-April to mid-May. Transplanting which is required with the use of seedling grafting approaches could be used for preventing or alleviating early season chilling stress^6^. Cold soil problem occurred in earlier spring sowing might be alleviated using grafting with superior rootstocks and transplantation. Because cotton is a tropical plant and some reports suggest that cold and wet soils increase disease susceptibilities; therefore, seedling grafting shows great promise to improve cotton yields in temperate-tropical production areas when using superior, cold tolerant and/or disease resistant rootstocks. Also transplanting could make wheat–cotton intercropping system possible by planting cotton after winter wheat harvest^43^ in June, for instance in Turkey, and immediately transplanting 30–50 days old cotton seedlings in soil without tilling. It is reported that transplantation of cotton after the winter wheat harvest has become a more common planting pattern in some region of China^25,26^. When applied transplanting makes double cropping possible in Western and Southeastern Turkey, which are two major cotton and wheat production regions.

In the present study, investigation of DNA alterations was assessed using SSR markers, also widely known as microsatellites. Depending on the species and the primer pairs used, the number of SSR markers varied between one to four in *G. barbadense* and *G. hirsutum*^1^. Banding patterns of SSR markers indicated that there was no DNA alteration in terms of SSR expansion or reduction between the genomic DNAs extracted from seeds of control plants and grafted seeds agreeing with none graft-based DNA alteration previous report in grapevine^22^. We noted differences on the onset of flowering among the intraspecific and interspecific grafts of three cotton species, altered gossypol level between *G. barbadense* and *G. hirsutum* (unpublished data) probably due to involvement of epigenetic modification in some grafting types^41^. Previous studies reported graft-transmissible RNA gene silencing signals in both the upward^30^ and the downward direction^32^ in some grafting experiments^33^. Grafting approaches, II and IV produced callus at graft junction zone making these approaches suitable for experiments dealing with graft transmissible nuclear genome transfer for the formation of graft hybrid^9^. Lateral buds produced from the callus could be useful for further investigation seeking molecular trafficking between partners of grafted plants. Considerable amounts of studies documented mobility of DNAs, RNAs and proteins restricted to the contact zone between scion and stock. Lateral shoots could be used to detect transient or heritable changes or mobility of DNAs, RNAs and proteins^10,15,33^. Transplanting which is required with the use of seedling grafting approaches could be used for preventing or alleviating early season chilling stress^6^. Cold soil problem occurred in earlier spring sowing might be alleviated using grafting with superior rootstocks and transplantation. Because cotton is a tropical plant and some reports suggest that cold and wet soils increase disease susceptibilities; therefore, seedling grafting shows great promise to improve cotton yields in temperate-tropical production areas when using superior, cold tolerant and/or disease resistant rootstocks. In addition, transplanting older, grafted seedlings avoids the susceptible window of seedlings dying from diseases such as damping off, when they emerge from seed sown directly in the field.

In conclusion, we reported four grafting approaches that are compatible for intraspecific and interspecific grafting among three cultivated cotton species, which are the main source of cotton fibers produced worldwide. The use of scions and rootstocks without cotyledon leaves, type III, was found to be an efficient grafting approach. This approach produced active grafts with no lateral shoot formation and DNA alteration and was suitable for transplantation to field within one month. Grafting and transplantation of cotton enable us to get two ways of benefits: one is that better crop quality due to the grafting effects and second one is making double cropping such as winter wheat-cotton growing possible within the same year. On the other hand, because grafting enables horizontal exchanges of both RNA and DNA molecules between the grafting partners, grafting approach II and IV that resulted in lateral shoot formation would be very useful in designing experiments investigating genetic or epigenetic exchange or horizontal genome or gene transfer studies. Graft induced genetic transformation, may become an emerging concept for a new horizontal plant breeding method. The use and improvement of established grafting methods between different cotton species and incorporation of recent and advanced molecular tools will advance our understanding and utility of interspecific grafting.

## Materials and methods

### Plant materials and grafting

Seeds of cotton lines, “Texas Marker-1” (TM-1), belongs to *Gossypium hirsutum* L., “Pima 3–79”, belongs to *G. barbadense* L., (these lines were developed in College Station, TX and are available through GRIN-global or contacting the USA or possibly other cotton curators and their collections) and Maydos Yerlisi (MY) belongs to *G. herbaceum,* were sown in small pots (0.35 L) in a greenhouse, located in Antalya, Mediterranean coast of Turkey, in 2018. Three to seven days sowing intervals were used to obtain seedlings with similar physiological stage. Two weeks after sowing, seedlings with two-leaf stage (at emerging the first leaf for grafting type IV) were transferred into a laboratory, acclimated for three complete days before starting the grafting experiments. Four different modified cleft-wedge grafting approaches, termed as type I, II, III and IV, were used. Grafting consists of cotyledon node and cotyledon leaves retained on scions but not on rootstocks (type I), cotyledon node and cotyledon leaves retained on rootstocks but not on scions (type II), unattached cotyledon node and cotyledon leaves on scions and rootstocks (type III), and halved cotyledon node and retained single cotyledon leaf from rootstocks and scions (type IV).

In type I grafting, (Fig. 1a), seedlings used as scions (TM-1, Pima 3–79 and MY) were prepared by cutting the native shoot 3–4 cm below cotyledon node, retained cotyledon leaves along with the developing true leaves with the shoot apex. The cut end of the scion was prepared making a deep V-shape cut on the exposed end of shoot apex. Seedlings used as rootstocks (TM-1, Pima 3–79 and MY) were prepared by decapitating the shoot 3–4 cm below the cotyledon node. The end of the rootstocks was prepared by cutting vertically downward to a depth of 2–3 cm to have a deep ‘V’ shape so the cut end of scion would fit well. Prepared scion was inserted onto a rootstock until it fit securely. The graft region (union) was secured using wrapping material Parafilm M, (Bemis Company, Inc, Neenah, WI) in a spiral, beginning at the top, taking care to maintain alignment of rootstock and scion, and sprayed immediately with sterile water^12,16^.

In type II grafting, (Fig. 1b), scions (TM-1, Pima 3–79 and MY) were prepared by cutting 2–3 cm above the cotyledon node and the end of scions was prepared making a V-shape cut on the exposed end of shoot apex. Seedlings that were used as rootstocks (TM-1, Pima 3–79 and MY) were prepared by cutting at the node-hypocotyl axis vertically downward to a depth of 2–3 cm to have a deep wedge ‘V’ shape. A scion was inserted into cotyledonary node of a rootstock until it fit securely. The graft region was secured with Parafilm in a spiral, beginning at the top, taking care to maintain alignment of rootstocks and scions, and sprayed immediately with water^12,16^.

In type III grafting, (Fig. 2a), scions (TM-1, Pima 3–79 and MY) were prepared as in the type I but the cotyledon leaves were removed. Seedlings that were used as rootstocks (TM-1, Pima 3–79 and MY) were prepared as follow: the native shoot just 1 cm below the cotyledon node was removed and the cut end was split vertically to a depth of 2 cm. The scion was inserted into the base of the vertically split rootstock and closely wrapped with Parafilm and sprayed immediately with water^12,16^.

In type IV grafting, (Fig. 2b), young seedlings with the first true leaf started to emerge were used as scions and rootstocks (TM-1, Pima 3–79 and MY). Seedlings were cut transversely beginning 2 cm above and ending 2 cm below the cotyledon node keeping single cotyledon leaf attached. Scions and rootstocks prepared by transversely cross cuttings were joined and closely wrapped with Parafilm and sprayed immediately with water.

Grafted seedlings along with control seedlings (not grafted) were immediately placed in a plastic growth tunnel (1 (W) × 2 (L) × 0.65 (H) m) exposed to light using 4 cool-fluorescence lamps (placed 10 cm above the tunnel corresponding 1 m above the apex of seedlings) and 4 cool-fluorescence lamps (2 m above the tunnel). Temperature of the plastic tunnel was adjusted to 28 °C under a 16:8 day/night photoperiod for 15 days. During the first 3 days, humidity was kept by water spraying in every 4 h during the first day and one spray was applied at midnight. After day 3, water spraying gradually decreased so that grafts received one spray in the fifteenth day.

Healthy and actively growing grafts and control seedlings were transferred to a greenhouse and transplanted in space of 0.35 × 0.75 m in single ridge row. Three replicates were made, each of which consisted of four seedlings in a completely randomized design. Three days before grafting and five days after grafting experiments, seedlings received 10 mL fertilizer solution [sodium dihydrogen phosphate, NaH_2_PO_4_, (2 mM), potassium nitrate, KNO_3_, (6 mM), calcium chloride, CaCl_2_.2H_2_O, (4 mM), ammonium nitrate, NH_4_NO_3_, (2 mM), magnesium sulfate, MgSO_4_.7H_2_O, (1 mM), boric acid, H_3_BO_3_, (2.5 µM), manganese sulfate, MnSO_4_.H_2_O (2 µM), zinc sulfate, ZnSO_4_.7H_2_O, (2 µM), copper sulfate, CuSO_4_.5H_2_O, (0.5 µM), sodium molybdate, Na_2_MoO_4_.2H_2_O, (0.33 µM) and ethylene-diamine di-2-hydroxyphenyl acetate ferric, Fe-EDHA, 5.7% Fe, (0.65 µM)].

Seedlings received conventional cotton growing practices such as weed control, irrigation and fertilizers^1^. Two different compound fertilizers differed in N, P_2_O_5_, and K_2_O contents were used. A fertilizer [20% N 20% P 20% K] was applied twice one month before flowering and at the beginning of flowering. Other fertilizer [15% N 30% P 15% K] was applied one month after flowering. Grafts and control plants received twice-chemical spraying for white flies, spiders and bollworms^1^.

### DNA extraction and marker studies

Five seeds each of which obtained from intraspecific and interspecific grafts along with their controls were used in DNA extraction studies. Genomic DNA samples were extracted from a single seed of each grafted and control sample using a DNA extraction protocol described in Karaca et al.^44^. DNA samples were evaluated using spectrophotometric and electrophoretic method as described in Ince et al.^45^. Simple sequence repeat markers (SSRs) were utilized as the detection tool of locus-specific DNA alteration. Ten SSR markers (MK004, MK011, MK017, MK020, MK021, MK028, MK044, MK055, MK072 and MK078) reported in Karaca and Ince^46^ were utilized. Polymerase chain reaction (PCR) amplification and agarose gel electrophoresis analyses were performed as described in Karaca and Ince^46^.

### Data collection and statistical analysis

Grafts at the second week of transplantation were scored as active growing healthy scion (successful grafting) or dead/dying scion (incompatible grafting) as survival rate. Numbers of true leaves were used to assess growth rate assuming the higher the number of produced leaves, the higher the growth rate during the fifth week of transplantation. Grafts were searched and counted for the presence of lateral shoot formation from the rootstocks.

Microsatellite DNA markers on gel images were scored for identification of DNA loci alteration. Presence or absence of a marker between a graft and its control counterpart was scored. Heterozygosity, defined as the proportion of sites on the chromosome at which two randomly chosen copies differ in DNA sequence, was calculated using formula *h* = 1 − ∑*x*^2^*i*, h = 1 − ∑xi^2^, where *x*_*i*_ is the frequency of the *i*th allele^47^. The value *h* reflects the underlying mutation (alteration) rate.

Collected data of survival rate, growth rate and lateral shoot formation were subjected to analysis of variance (ANOVA) and Tukey–Kramer HSD test utilizing JMP Statistical Discovery Software Version 8.0 (SAS, Cary, NC, USA). Because DNA marker data showed zero heterozygosity for all ten-microsatellite loci used, the data were not statistically analyzed.
